# Membrane and Protein Interactions of the Pleckstrin Homology Domain Superfamily

**DOI:** 10.3390/membranes5040646

**Published:** 2015-10-23

**Authors:** Marc Lenoir, Irina Kufareva, Ruben Abagyan, Michael Overduin

**Affiliations:** 1 School of Cancer Sciences, Faculty of Medical and Dental Sciences, University of Birmingham, Birmingham B15 2TT, UK; E-Mail: m.lenoir@bham.ac.uk; 2 Skaggs School of Pharmacy and Pharmaceutical Sciences, University of California, San Diego, 9500 Gilman Drive, La Jolla, CA 92093, USA; E-Mails: ikufareva@ucsd.edu (I.K.); rabagyan@ucsd.edu (R.A.); 3 Department of Biochemistry, Faculty of Medicine and Dentistry, University of Alberta, Edmonton, AB T6G 2H7, Canada

**Keywords:** bilayer insertion, lipid binding, membrane trafficking, MODA, peripheral membrane protein, PH domain, phosphoinositide recognition, plasma membrane, pleckstrin homology domain, small GTPase

## Abstract

The human genome encodes about 285 proteins that contain at least one annotated pleckstrin homology (PH) domain. As the first phosphoinositide binding module domain to be discovered, the PH domain recruits diverse protein architectures to cellular membranes. PH domains constitute one of the largest protein superfamilies, and have diverged to regulate many different signaling proteins and modules such as Dbl homology (DH) and Tec homology (TH) domains. The ligands of approximately 70 PH domains have been validated by binding assays and complexed structures, allowing meaningful extrapolation across the entire superfamily. Here the Membrane Optimal Docking Area (MODA) program is used at a genome-wide level to identify all membrane docking PH structures and map their lipid-binding determinants. In addition to the linear sequence motifs which are employed for phosphoinositide recognition, the three dimensional structural features that allow peripheral membrane domains to approach and insert into the bilayer are pinpointed and can be predicted *ab initio*. The analysis shows that conserved structural surfaces distinguish which PH domains associate with membrane from those that do not. Moreover, the results indicate that lipid-binding PH domains can be classified into different functional subgroups based on the type of membrane insertion elements they project towards the bilayer.

## 1. Introduction

The pleckstrin homology (PH) domain was discovered 22 years ago in various proteins including pleckstrin which are involved in signaling, cytoskeletal organization, membrane trafficking and phospholipid processing [1,2]. The elucidation of the three dimensional structure of this 100 residue module revealed its characteristic seven stranded antiparallel β-sheet and C-terminal α-helix [3]. The phosphatidylinositol-4,5-bisphosphate binding function of some PH domains was soon discovered [4]. This finding indicated that some PH domains are able to transiently anchor various proteins to intracellular membrane surfaces, and suggested that they could help to recruit cytosolic proteins to organelle surfaces [5]. However, it subsequently turned out that most yeast PH domains do not bind specifically to phospholipids and do not recruit proteins to membranes [6]. Moreover, other PH domains associate with G-protein coupled receptors [7], allow Dbl Homology (DH) domains to regulate Rho-family GTPase exchange Factors (GEFs) [8], or are found in Ran-binding domain proteins [9], thus indicating the role of a PH-like superfold as a universal module mediating protein-protein and protein-membrane associations. PH-like folds are found in the C-terminal part of the FERM domain (a clover-like structurally conserved protein module involved in localizing proteins to the plasma membrane), several phosphotyrosine peptide binding domains (scaffolding modules that recognize phosphoinositides along with phosphorylated or non-phosphorylated peptides), Ran binding domains, the N-terminal p62 subunit of TFIIH, WH1/EVH1 domains (recognizing proline-rich peptides), and GRAM domains (an intracellular protein- or lipid-binding domain found in glucosyltransferases and myotubularins). Furthermore, disease-linked mutations within membrane binding sites of some PH domains [10] indicate a critical functional importance, justifying a more comprehensive study.

In order to predict which members of the PH domain superfamily bind membranes, we applied the MODA program that has been trained to identify membrane binding surfaces in any high resolution protein structure [11]. We focused on a subset of the PH domain superfamily that is annotated as “plekstrin homology (PH) domain” in the latest release of the Uniprot database. Analysis of the available structures for these domains revealed unprecedented functional surfaces that are considered in light of the available biological and biochemical data. This analysis indicates that at least 61% of annotated PH domains associate with membranes, and present divergent features that account for their distinct specificities and affinities. The tools and data can be used to classify functional properties and consequences of deleterious mutations across the superfamily.

## 2. Experimental Section

### 2.1. Alignment and Conservation

Protein sequences that contain at least one PH domain were selected from the curated SwissProt database. Only one isoform per set of entries was chosen, and the PH domains of the selected proteins were extracted for alignment. In total, the September 2015 release of the Uniprot database contained annotated PH domains in approximately 285 distinct human proteins. The alignment was solely based on the sequences of the PH domains, and the phylogenetic tree was constructed accordingly. The sequences were aligned using Multiple Alignment using Fast Fourier Transform (MATFF) and subsequently used to build a phylogengetic tree within phyML [12]. The conservation across the PH domain family was visualized using iTOL [13] where the structural domains of the protein, as annotated in the Uniprot database, were represented. The sequence alignments were analyzed within Ugene [14].

### 2.2. Predication of Membrane Binding Sites

The coordinates of the PH domains were obtained from the Protein Data Bank and submitted to the MODA algorithm [11]. When protein complexes or multimeric proteins were found, only the structure corresponding to the PH domain was analyzed. Ensembles of NMR structures were considered to predict a membrane binding site when at least half of the conformers exhibited the patch. By MODA patch, we mean at least two consecutive residues that are predicted to mediate membrane interaction. The experimental validations of binding sites were collected from studies that reported direct binding interactions *in vitro*. This was also intended to minimize the potential for indirect interactions that could arise from analyses of cellular data or complex systems. Predicted membrane binding was considered to be validated when experiments were carried out with membrane mimics such as phosphatidylinositolphosphate (PIP) ligands, micelles, bicelles, or liposomes using methods including NMR, X-ray crystallography, fluorescence, or dot-blot assays.

## 3. Results

### 3.1. PH Domains, Similarities and Differences

**Figure 1:**
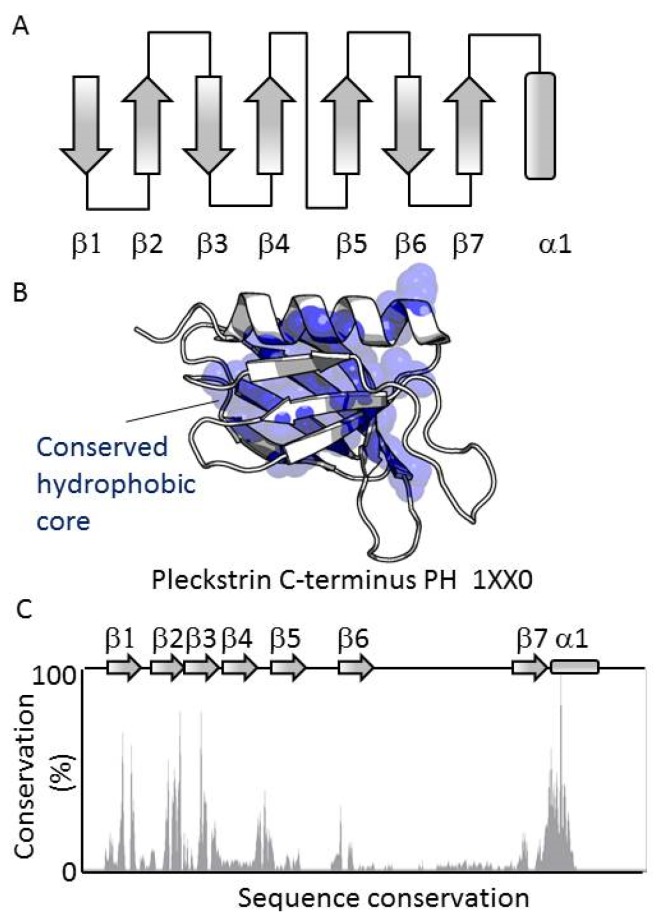
Structure of the PH domain. (**A**) The secondary structure elements of the PH domain fold are shown, including the seven antiparallel β-strands and the C-terminus helix; (**B**) Mapping of residues with at least 50% identity across the PH domain family. The conserved residues are represented by yellow spheres; (**C**) Sequence conservation within the human PH domains. The conservation is expressed in percentage of conservation according to the ClustalX scheme. The reference sequence is that of the C-terminal PH domain of pleckstrin.

All members of the PH domain superfamily contain a conserved fold based on seven antiparallel β-strands arranged in a sandwich which is capped at its splayed corner by a C-terminal α-helix. These elements form a central hydrophobic core that stabilizes the consensus structure (Figure 1A–C). The most conserved elements are the hydrophobic residues of the secondary structures that contribute to the hydrophobic core of the domain. The central Trp of the helix is the most conserved residue, displaying 98.2% identity, with its mutation resulting in misfolding. The conserved secondary structure elements contrast with the much more variable loops which connect the β-strands (Figure 1D). The low conservation of the composition and length of the exposed, dynamic loops reflects the range of unique roles of the array of PH domains, and present challenges for accurately predicting their functions.

**Figure 2:**
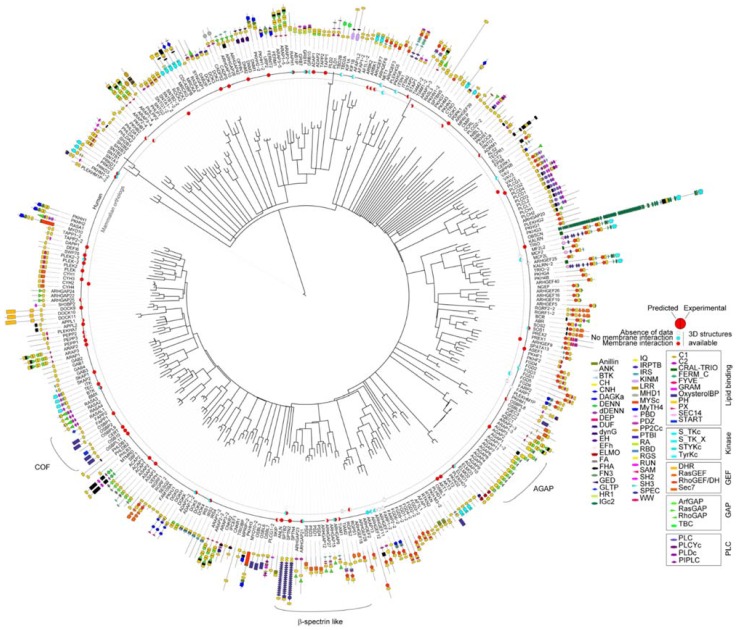
Diagram of the relatedness of human proteins based on PH domain sequence similarity. The tree was built for the human PH domains with distances indicating similarities using Itol [13]. For each PH domain, the modular architecture is indicated and the length of the protein is represented by a line whose inner extremity corresponds to the start of the protein. For proteins containing several PH domains, each PH domain is numbered according to its relative position within the protein. Deposited PH domain structures from human and mammalian sources are indicated by a disc that is colored to indicate the experimental and predicted membrane binding functions. Experimental evidence of the binding was considered only when the role of the PH domain was specifically examined rather than the entire protein. Protein domains are grouped by functions, GAP, GEF, kinase/phosphatase and membrane binding. Abbreviations of the protein domains are indicated in the Table A1 [4,10,15,16,17,18,19,20,21,22,23,24,25,26,27,28,29,30,31,32,33,34,35,36,37,38,39,40,41,42,43,44,45,46,47,48,49,50,51,52,53,54,55,56,57,58,59,60,61,62]. The abbreviations of the domains are given in Table A1.

To inspect the PH domain superfamily as a whole and understand how its domains are involved in diverse biological functions, we gathered the amino acid sequences of 334 human PH domains. They are present in 285 distinct proteins some of which contain up to 5 PH domains, such as ARAP members. Only 13% of these multifaceted proteins contained more than one PH domain, and most of these have two (9%). Based on multiple-sequence alignments, a phylogenetic tree was built by maximum likelihood for the PH domain superfamily (Figure 2). Several sub-families could be distinguished such as the COF family that contains OSBP, CERT and FAPP proteins or β-spectrin-like (Figure 2). The sequence similarities of the PH domains are often mirrored by the overall function of their host protein such as Arf-GARP (AGAP) or clustered pairs, for example, of DH-PH domains. This suggests that despite the evolutionary changes, sections of the protein sequence have been maintained for functional purposes. For instance, FARP or FGD proteins contain two PH domains, each carrying a specialized role (Figure 2). The DH domain of FARP1 is followed by two consecutive PH domains separated by unfolded regions. The first one contains a putative PIP binding site whereas the second autoinhibits the activity of the DH domain by folding back and blocking its interaction site [15].

Comparison of protein domain architectures alongside the phylogenetic tree (Figure 2) also highlights a recurring role of PH domains in membrane-associated events mediated by small GTPase members. These proteins also contain guanine exchange factor (GEF, 79 proteins) or GTPase-activating protein (GAP, 73 proteins) functions, lipid carriers (11, proteins including oxysterol binding domain, START or GLTP) or kinase domains (19). This presence of PH domains in so many small GTPases is in accordance with the accumulated evidence that suggests that PH domain act as major regulators of these membrane-associated signaling switches [63]. In total, PH domains are found with DH domains in 62 proteins, with RasGEF in 6, RhoGAP in 21, ArfGAP in 2, RasGAP in 8, and Sec7 in 11 proteins. A total of 43% proteins of the PH superfamily contain at least one these GTPase regulating modules, indicating the potential importance of PH membrane interactions to this additional signaling field.

### 3.2. Genome-Wide Analysis of Membrane Binding of PH Domains

The membrane interactions of 69 human PH domains and 19 mammalian orthologues were analyzed using available structural and experimental data. This yields a total of 88 cases that together represent 26% of the PH superfamily (Figure 2).

**Figure 3:**
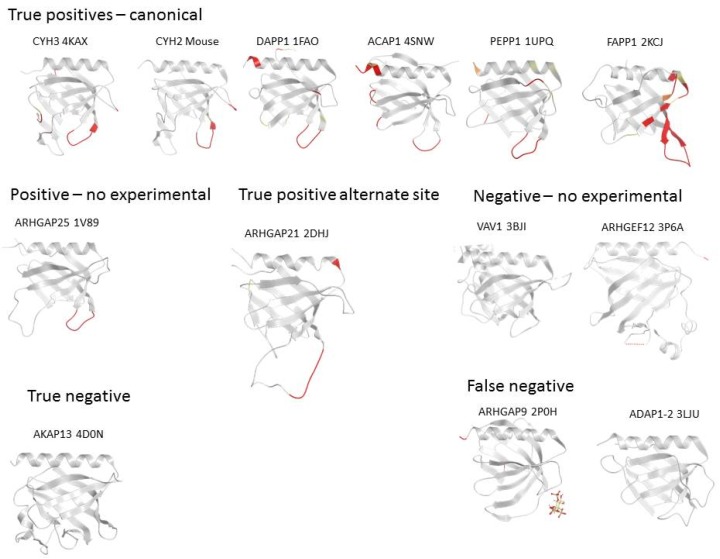
Examples of PH domain structures showing the membrane interaction sites that are predicted by MODA. The name of each protein is followed by the PDB deposition number and is indicated above each structure. The predicted membrane interacting sites are shown in a gradient of red-orange-yellow [11] and missing segments are represented by dotted lines.

In each case, the membrane interactions sites were calculated using MODA (Figure 3), a molecular analysis software tool using residue fragment level approximation and statistically trained weights to detect likely membrane-interacting patches on protein structures [11]. MODA was developed using the ODA algorithm as a foundation [64] and then trained to identify membrane binding sites. The experimental validation of each predicted membrane binding site is indicated along the inner circles in Figure 2. This shows that a total of 67 PH domain structures are likely to bind membranes based on *in silico* and *in vitro* binding assays, while 21 PH domain structures lack obvious membrane binding features or abilities.

In particular, MODA predictions of membrane binding PH domains were confirmed experimentally for 36 out of the 53 positive cases [16,17,22,23,26,27,30,32,33,35,41,44,45,47,50,53,56,58,59,65,66,67,68,69]. Similarly, of the 49 domains found to interact with membranes experimentally, 36 were confirmed by MODA predictions. Discrepancies between the experimental and computational results occurred where MODA falsely predicted that a PH structure did not bind membranes (10 cases) or predicted a membrane-binding domain where experimental data argued otherwise (3 cases).

Detailed structural analysis of the false negatives indicates that in most cases MODA failed to predict a known membrane binding site due to structural factors. For example, some structures lack density for exposed hydrophobic residues in known binding loops, or contain an element that is too constrained to allow a predictable membrane interaction such as in the ADAP1-2, IRS1, GRB14, and RAPH1 proteins. For instance, the membrane binding site of ADAP1-2 was co-crystallized with a phosphoinositide headgroup [56] but was not predicted to bind bilayers by MODA. The structure of the protein shows that the β5-β6 and β1-β2 loops where the headgroup binds are close in space and drawn into the protein core in a conformation that precludes bilayer insertion. This case could reflect a singular structural state or a functional anomaly where the PH domain does not need to insert into a membrane to recognize the lipid headgroup.

The false positives generated by MODA include PRKD2 or PRKD3 where the β3-β4 loop or the β5 strand were identified as possible membrane interacting sites. However, these sites actually interact directly with the kinase domain that they are known to regulate, although we cannot formally exclude the possibility that lipids may also bind transiently here within the cell.

Definition of which PH domains do not associate even transiently with any membrane is challenging. There is a scarcity of studies reporting the lack of interaction between PH domains and membranes for obvious reasons. The experiments needed to disprove membrane binding are demanding, in particular when the affinity is typically in the μM-mM range and there are many potential lipid ligands and membranes. Moreover there is generally little appetite for publishing negative results. Nonetheless, the absence of membrane interactions is experimentally established for four proteins: AKAP13, ARHGAP21, PRKD2, and PRKD3, with the predicted lack of membrane binding by AKAP13 being in agreement with recent results [70]. Given the small number of such negative test cases, we investigated two proteins further. In particular, we demonstrated aqueous solubility rather than lipid binding by the wild-type PRKD1 PH domain and a FAPP1-PH domain containing Y11E and L12E mutations in its membrane insertion motif. In both cases MODA analysis of models based on their high degree of similarity with known structures showed that they lacked membrane binding signatures. This prediction was fully consistent with our bilayer and lipid binding assays by ^1^H, ^15^N resolved NMR spectroscopy showing a lack of membrane interactivity (data not shown).

Interestingly, the RhoGEF subfamily members generally exhibit a PH domain that have no membrane binding signatures and are positioned immediately after the DH domain. Such cases include AKAP13, ARHGEF3, ARHGEF11, ARHGEF12, FGD3, FGD5, ITSN1, MCF2L, NET1, and VAV. No accurate prediction could be achieved by MODA with the related PH domains of FARP1-2 or FARP2-2 due to the lack of sufficient resolution, although a structured interaction of the C-terminal PH domain of FARPs with the DH domain is unlikely [15]. In contrast, other members of this PH subfamily including ARHGEF6, ARHGEF7, ARHGEF9, ASEF1, TRIO, and TIAM1-2, as well as SOS1 appear by MODA to interact with membrane but due to their juxtaposition with a DH domain and role in regulating the GEF activity are more likely to be involved in protein-protein interactions. Together this demonstrates the value of considering familial patterns when interpreting MODA results, which in turn can pinpoint key residues to mutate and test as ligand specificity determinants.

### 3.3. Comparison of Membrane Interactions Designates the β1-β2 Loop as the Canonical Binding Element

**Figure 4:**
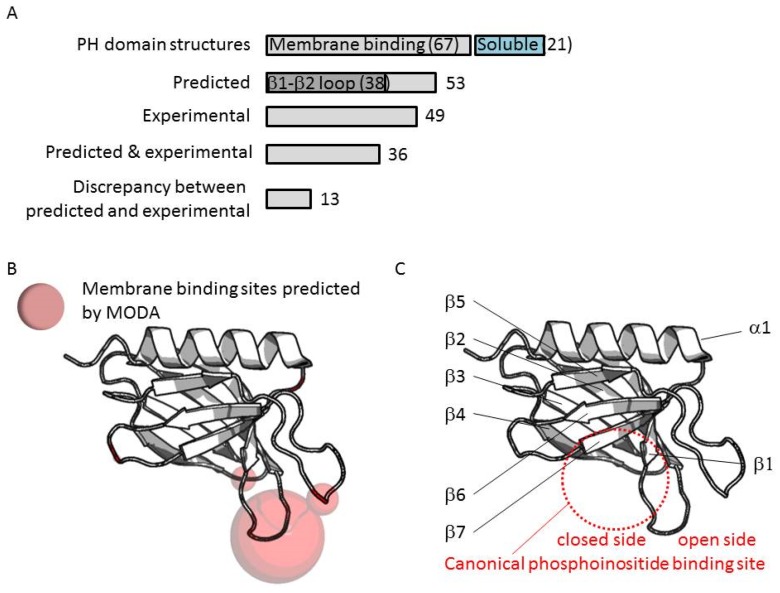
(**A**) Recurrent membrane interactions sites predicted by MODA in PH domain structures; (**B**) The volume of the sphere shown on the ribbon structures is proportional to the number of sites identified herein; (**C**) The fold of the PH domain with the variable regions is depicted with the secondary structures indicated.

In total 88 PH structures were analyzed, although poor resolution thwarted prediction from 11 structures. Out of the remaining 77 structures, membrane interaction sites were predicted for five domains, 38 of which contained a binding patch at the β1-β2 loop (Figure 4A). Comparison of phosphoinositide binding modes here indicates that two binding sites can be distinguished on either side of the loop. Most binding take place at the “inner” or “closed” side of the loop whereas a few β-spectrin-like structures have been co-crystallized with inositol headgroups positioned at the “open” side of the loop (Figure 4C). Such non-canonical binding events are displayed by the PH domains of the ARHGAP9, ARHGAP27, RGPS1, SPTB2, SPTN2, TIAM1, and TIAM2 proteins.

**Figure 5:**
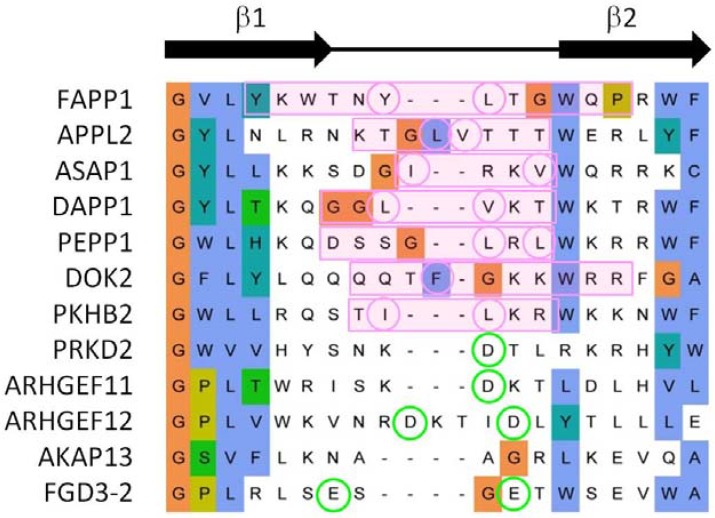
Sequence alignment of representative β1-β2 loop of PH domains. The MODA patch is indicated with a pink box and the hydrophobic amino acids circled in pink. When no MODA patch was found, the acidic residues are circled in green. The sequence alignment is extracted from that of the entire family as described in the Experimental Section.

In summary, our genome-wide analysis indicates that the exposed β1-β2 loop represents the primary bilayer interacting element in 70% of those PH domains which associate with membranes. Our previous studies of the FAPP1 protein [71,72] revealed that it is the tip of this loop that mediates both phosphoinositide-specific binding and non-specific insertion into membrane bilayers. Alignment of PH domain sequences shows that both basic and proximal hydrophobic residues are present in membrane binding cases, whereas more acidic and fewer hydrophobic residues are found in non-binders (Figure 5). Thus these elements are useful discriminators for sequence-based prediction of canonical membrane binding by PH domains even in the absence of a 3D structure.

### 3.4. Extrapolation of Membrane Interaction Predictions

The results of MODA predictions can be translated into the discovery of novel membrane interactions sites, and can drive the design of mutations or experiments to gain insight into protein functions. Through the following examples, we inspect the consistency of predictions of membrane binding across the family.

### 3.5. Four Phosphate Adaptor Protein 1 (FAPP1) as a Paradigm of Canonical Membrane Binding

The PH domain of FAPP1 illustrates a case where the prediction is fully consistent with the binding site determined experimentally. Extensive biophysical, structural and computational data [71,72] support the presence of the membrane binding patch mapped by MODA to residues Tyr6 to Pro17. The NMR experiments and mutagenesis clearly show that the insertion of the exposed Tyr11 and Leu12 sidechains extends into the hydrophobic core of the bilayer. The rest of the loop stabilizes the interaction by forming hydrogen bonds and through shallow insertion into the membrane headgroups [71]. Residues of the β1-β2 loop also form an extended and solvent-exposed segment in related structures such as those of CERT, OSB10, or OSBPL11. In all these structures, the region that surrounds the two hydrophobic residues of the tip are also predicted to interact with membranes but not the entire phosphoinositide binding site, which contains portions of the β3, β4, and β7 strands. Beyond these closely related proteins, other PH domains that fall into this pattern with two consecutive hydrophobic residues at the extremity of β1-β2 loop include ACAP1, DAPP1, PEPP1, and SWP70 (Figure 3). However the reciprocal is not always true, as alone these hydrophobic residue pairs are not sufficient to guarantee the presence of a genuine membrane interaction site.

### 3.6. ARHGAP9 and Discrepancies between Predictions and Experimental Validations

Besides the PIP canonical binding site, a sub-family represented by β-spectrin-like proteins hosts a PIP binding site at the “open side” of the β1-β2 loop [17]. This noncanonical binding site is formed by cationic side chains that point towards the start of the C-terminal helix, and are supported by additional cationic residues within β5-β6 loop that provides complementary contacts [17] rather than the β7 strand used in the canonical binding mode. Predictions of the membrane binding sites are poor for this sub-family, and fail to predict membrane binding sites in the cases of ARHGAP9 or SPTB2. Such proteins contain no exposed hydrophobic loop by the PIP binding site. The structure of ARHGAP9 co-crystallized with a phosphoinositide headgroup suggests that the PH domain interaction with membrane remains superficial, no residue being capable of inserting deeply into the bilayer. Insulin receptor substrate 1 protein (IRS1) also possesses residues compatible with phosphoinositide binding, however, based on our prediction the interaction of IRS1 seems limited to the headgroup of the PIP that it binds rather than indicating insertion into the membrane.

### 3.7. Evidence for an Alternate Binding Site in ARHGEF9

The regulation of the ARHGEF9 protein, which is also known as Collibistyn I, is dominated by the relative position of its domains. The activity of its DH-PH tandem domain is largely controlled by the position of its SH3 domain, which binds the DH domain and prevents the PH from interacting with membranes [73]. Once the autoinhibition releases the PH domain of ARHGEF9, it interacts with PIP and membranes using a hydrophobic patch located at the tip of the β3-β4 loop [74]. Its β1-β2 loop strongly interacts with a long β6-β7 loop, closing the canonical site of PH domains. This site was predicted by MODA for AHRGEF9 but also for the related PH domain of ASEF1. The convergence of the prediction, which is solely based on the structure, demonstrates how MODA can be used to pinpoint areas of the protein that may be otherwise overlooked in sequence alignments.

### 3.8. A-Kinase Anchoring Protein 13 (AKAP13) and the Absence of Membrane Interaction

AKAP13 remains one of the few examples of PH domains for which the absence of membrane interaction has been determined experimentally [70]. The absence of MODA patch correlates the observations. Other examples include ARHGAP21 which does not recognize PIPs based on liposome sedimentation assays [62]. However, it is important to note that this technique is most effective at showing strong interactions, it may fail to detect weak or transient interactions that are commonly mediated by some peripheral membrane proteins.

## 4. Discussion

The interactions of peripheral proteins with membranes range over a wide spectrum of affinities ranging from nM to mM. This is consistent with the biological roles of initial sampling of surfaces, specific recognition of lipid ligands, tight anchoring of multi-subunit assemblies on membrane, and dynamic transfer of cargo. While a tight binding event is easily confirmed with most experiments, the discovery of weak interactions proves more challenging and requires more sensitive approaches.

Moreover, it is common to observe that the lipid specificity contains a method-dependent component which complicates the analysis and interpretation of the binding. For instance, the use of dot-blots or PIP strips on complex surfaces generates results which do not necessarily agree with solution data from NMR, fluorescence data with modified proteins, or surface plasmon resonance (SPR) data from sensor chips. Similarly, excessive addition of anionic lipids can systematically increase the affinity of some proteins for membrane. Here, MODA focuses on the inherent membrane binding properties, regardless the lipid specificity. We show that it provides a useful tool for discovering membrane interaction sites and allows discrimination of binding modes within a family for validation by virtually any experimental tool.

This study sheds a new light onto the PH domain family, comparing the sequences, structures and membrane binding capabilities of a large number of PH domains. First, it showed the striking number of GEF or GAP domains associated with PH domains which are often situated proximally and regulate their activities.

The second observation derived from this genome-wide analysis of PH domains is that the number of PH domains that bind membranes is far greater than those that do not. Current estimates places the fraction of PH domains that bind membranes to only about 20%, while our analysis of a well-characterized quarter of the PH proteome shows that at least 61% of the members bind membranes. Moreover, in light of their mechanisms, it is now feasible to test how PH domain interactions contribute to, for example, membrane limited GTPase reactions, as well as assembly of and signaling from membrane-associated complexes. Importantly, such sites can be exploited for therapeutic intervention, as demonstrated by the identification of inhibitors directed at the membrane binding PH domain of oncogenic Akt [75,76,77,78,79].

This analysis resolves whether protein superfamilies possess conserved features that can be used to distinguish cytosolic and peripheral membrane states, thus addressing a long-standing debate. In particular, previous postulations that primary sequence motifs can predict lipid binding have proven tenuous, and here we show that 3D signatures are genuinely predictive. While some 3-phosphoinositide binding PH domains appear to exhibit a common motif [65], the sequence highlighted has since suffered numerous exceptions, thus limiting their predictive value. More useful than the sequence alone is the distinctive surface that encompasses the membrane binding entity [26] and is often supported by an electrostatic dipole in the protein [80]. Altogether this suggests that it is not possible to reliably predict membrane interactions based on the sequence only. Rather, the availability of a 3D structure-based predictive tool such as MODA, which is available at moda.ucsd.edu for free, allows interrogation of individual proteins as well as genome-wide studies.

A note of caution is warranted. The definition of a membrane binding domain may vary with the case under investigation. Weak binding may not be detected by most techniques and therefore, when the analysis is carried out, a genuine transient or non-specific membrane binding domain may be regarded as a non-binder, introducing a bias towards PH domains that are tight binders or required a specific lipid ligand. Nonetheless, weak binding events predominate in nature, and often contribute to multivalent interactions and multi-step assembly processes. Sensitive tools to detect and allow the characterization of even weakly binding domains are needed to understand the execution and regulation of cellular events. Here this capability has been demonstrated with a large superfamily for the first time, and should allow discovery of many more biological and regulatory functions in other proteins.

## Appendix

**Table A1:** List of abbreviations corresponding to the protein domains that are shown in Figure 2.

Abbreviation	Name
ANK	Ankyrin repeats
BTK	Bruton’s tyrosine kinase Cys-rich motif (Zn^2+^)
C1	Protein kinase C conserved region 1 (C1) domains (Cysteine-rich domains)
C2	Protein kinase C conserved region 2 (CalB)
CH	Calponin homology domain
CNH	Domain found in NIK1-like kinases, mouse citron and yeast ROM1, ROM2
CRAL-TRIO	cellular retinaldehyde-binding protein and TRIO guanine exchange factor domain
DAGKa	Diacylglycerol kinase accessory domain (presumed)
DENN	Differentially expressed in neoplastic *versus* normal cells
DEP	Domain found in Dishevelled, Egl-10, and Pleckstrin
DUF	Domains of unknown function
dynG	Dynamin-type guanine nucleotide-binding (G)
EFh	EF-hand, calcium binding motif (Ca^2+^)
EH	Eps15 homology domain
ELMO	EnguLfment and cell Motility
FA	FERM adjacent
FERM	Ezrin/radixin/moesin
FERM_C	FERM C-terminal PH-like domain
FHA	Forkhead associated domain
FN3	Fibronectin type 3 domain
FYVE	Protein present in Fab1, YOTB, Vac1, and EEA1
GED	Dynamin GTPase effector domain
GLTP	Glycolipid transfer protein
GRAM	Domain in glucosyltransferases, myotubularins and other putative membrane-associated proteins
HR1	Rho effector or protein kinase C-related kinase homology region 1 homologues
IGc2	Immunoglobulin C-2 Type
IQ	Short calmodulin-binding motif containing conserved Ile and Gln residues.
IRPTB	IRS-type PTB
IRS	Phosphotyrosine-binding domain
KINM	Kinesin motor domain
LRR	Leucine-rich repeats, outliers
MHD1	Munc13 homology domain
MORN	Membrane Occupation and Recognition Nexus
MYSc	Myosin. Large ATPases
MyTH4	Domain in Myosin and Kinesin Tails
Oxysterol_BP	Oxysterol binding protein
PBD	P21-Rho-binding domain
PDZ	Domain present in PSD-95, Dlg, and ZO-1/2
PH	Pleckstrin homology
PIPLC	Phosphatidylinositol-specific phospholipase C Y domain
PP2Cc	Serine/threonine phosphatases, family 2C, catalytic domain
PTBI	Phosphotyrosine-binding domain (IRS1-like)
PX	Phox homology
RA	Ras association (RalGDS/AF-6) domain
RAS	Ras subfamily of RAS small GTPases
RBD	Raf-like Ras-binding domain
RGS	Regulator of G protein signalling domain
RUN	Domain involved in Ras-like GTPase signalling
SAM	Sterile alpha motif
SEC14	Domain in homologues of a S. cerevisiae phosphatidylinositol transfer protein
SH2	Src homology 2 domains
SH3	Src homology 3 domains
SPEC	Spectrin repeats
START	StAR-related lipid-transfer
TBC	Domain in Tre-2, BUB2p, and Cdc16p—probable Rab-GAP
uDENN/dDENN	Upstream/downstream DENN
WW	Domain with 2 conserved Trp (W) residues
